# 

**Published:** 1872-12

**Authors:** 


### PART III.

### Editorials, Correspondence and Miscellaneous.

☞ Should any of our Subscribers or Exchanges fail to get THE COMPANION regularly, they will confer a favor by notifying our Business Manager of the fact.

☞ Our Business Manager will faithfully forward THE COMPANION. Subscribers will please notify Post-masters that it will reach their offices, if not lost on the way, between the 15th and 20th of every month. Should it fail to come, after a reasonable time, notify the Business Manager, and it will be sent.

☞ We respectfully solicit Original Articles, Reports of Societies, Clinics Home and Foreign Correspondence, News, etc., etc., of interest to medical readers.

☞ To insure publication, articles should be practical, brief as possible, and carefully written, on ONE side of the paper. Articles should be received by the 15th of one month to insure publication in the next. ☞ Friends, send in your articles. ☜

☞ All Subscribers and others will please write their names, post-offices, county and State *plainly*. ☞ *Remember, this is now required by the Post-Master General*. All business letters, in future, should be directed to Charles Whitehead, Business Manager.

### TO OUR SUBSCRIBERS.

I have made arrangements to take charge of the Business Department of the GEORGIA MEDICAL COMPANION. My best efforts in its behalf, my personal attention to my department, in the prompt mailing of the journal, in dispatching of business letters upon reception, in receipting promptly for moneys sent me, and my sincere regard for the wishes of subscribers in particular, is promised.

By the arrangement between the Editors and myself, I become the agent of every subscriber, and I appeal to each one to act as my agent, and send me one or more subscribers, which I entreat of them, with the promise on my part, that they shall receive all courtesy and attention at my hands. My endeavors will be to make our relations pleasant and satisfactory; and, for the Editors, I earnestly request the friends of the COMPANION to aid them, that the wants, interests and wishes of a noble profession may be fully and completely represented. The names of new subscribers should be forwarded at once.

In connection with the Business Department of the COMPANION, I have established a PURCHASING AGENCY and solicit orders for purchases in any department of business. My facilities are superior, and especially in Surgical Instruments, Books, Drugs and Physicians' Supplies. Cash must accompany all orders.

CHARLES WHITEHEAD, Box 324, Atlanta, Ga.

